# Effects of Structure and Constituent of Prussian Blue Analogs on Their Application in Oxygen Evolution Reaction

**DOI:** 10.3390/molecules25102304

**Published:** 2020-05-14

**Authors:** Dongni Zhao, Yuezhen Lu, Dongge Ma

**Affiliations:** 1 School of Science, Beijing Technology and Business University, Beijing 100048, China; celinazhao07@gmail.com; 2 Department of Engineering, Lancaster University, Lancaster LA1 4YR, UK; lvyuezhen1@gmail.com

**Keywords:** oxygen evolution reaction (OER), Prussian blue analogs (PBA), electrocatalysis, photoelectrocatalysis, nanostructure of catalyst, green catalyst, metal–organic framework

## Abstract

The importance of advanced energy-conversion devices such as water electrolysis has manifested dramatically over the past few decades because it is the current mainstay for the generation of green energy. Anodic oxygen evolution reaction (OER) in water splitting is one of the biggest obstacles because of its extremely high kinetic barrier. Conventional OER catalysts are mainly noble-metal oxides represented by IrO_2_ and RuO_2_, but these compounds tend to have poor sustainability. The attention on Prussian blue (PB) and its analogs (PBA) in the field of energy conversion systems was concentrated on their open-framework structure, as well as its varied composition comprised of Earth-abundant elements. The unique electronic structure of PBA enables its promising catalytic potential, and it can also be converted into many other talented compounds or structures as a precursor. This undoubtedly provides a new approach for the design of green OER catalysts. This article reviews the recent progress of the application of PBA and its derivatives in OER based on in-depth studies of characterization techniques. The structural design, synthetic strategy, and enhanced electrochemical properties are summarized to provide an outlook for its application in the field of OER. Moreover, due to the similarity of the reaction process of photo-driven electrolysis of water and the former one, the application of PBA in photoelectrolysis is also discussed.

## 1. Introduction

With the rapid development of global industrialization, the demand for sustainable energy has become an urgent need. About 80% of the energy currently used for humans comes from fossil fuels, but this form of energy has limited reserves, and its combustion products have undesirable negative impacts on the environment [1,2]. As a result, scientists are devoted to developing innovative technologies to convert and store different types of green energy, and thereby the goal of cost reduction, sustainable development, and improved eco-safety could be further achieved.

A significant prerequisite for the application of green energy is the development of efficient energy conversion systems [3,4]. Electrochemical methods, such as the electrolysis of water, rechargeable metal–air batteries, and fuel cells, have attracted the attention of researchers resulting from their feasible excellent performance in various aspects [5]. However, the restriction of their development can mainly be attributed to the harsh reaction conditions to overcome the high kinetic barrier of hydrogen evolution reaction (HER), oxygen evolution reaction (OER), and oxygen reduction reaction (ORR) [4,6,7]. Therefore, the bright prospects of these devices will benefit from the exploration and investigation of appropriate electrocatalysts [8,9].

Besides being an essential semi-reaction in water splitting, OER also has significance for generating molecular oxygen. Oxygen is not only necessary for living, but also has versatile utilization in various industries. Molecular oxygen is supposed to be the most commonly used oxidant in the energy sector, acting as the electron acceptor during fuel combustion and making the chemical bonds in fuel molecules more vulnerable to combustion and releasing energy. At present, the industrial production approach of obtaining high-concentration oxygen is mainly to separate or purify air, but such methods are difficult to achieve sustainability due to the increasing demand for in-put of energy [10].

**Figure 1:**
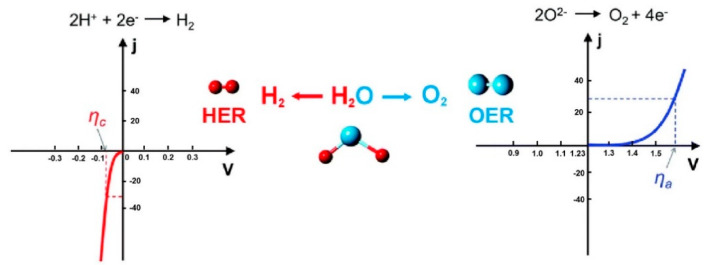
The polarization curves of HER (left) and OER (right). $\eta_{c}$ and $\eta_{a}$ represent the overpotential at the same current density (j) required from HER and OER, respectively. Adapted with permission from [13]. Copyright © 2017, Royal Society of Chemistry.

Considering the above factors, accelerating the development of OER is urgent. Simultaneously, among them, OER can be regarded as one of the most complicated processes because of the complexity and irreversibility of its reaction properties, as well as the extremely low current density near the equilibrium potential [11,12]. Figure 1 illustrates the polarization curves of HER and OER, and it can be seen that OER requires higher overpotential than HER to reach the same current density, which means that the kinetic barrier of OER is harder to overcome, and this phenomenon could be ascribed to the transportation of more electrons during OER [13]. Currently, commercial OER catalysts are mainly noble-metal compounds such as IrO_2_ and RuO_2_ [14,15], but their scarcity has driven up their prices. Thus, it is of irreplaceable importance in industrial production to design novel catalysts with excellent sustainability to surmount the kinetics barrier and accelerate the reaction rates.

The development of OER catalysts is assumed to follow three general directions: first, clarify the specific active sites in the reactions by in-situ characterization techniques [15]; second, design catalytic architectures with facilitated accessibility [16]; and, third, scale them up into devices [8]. The second point is particularly significant for materials researchers, and is mainly discussed in this article.

Prussian blue (PB) was initially fabricated through an unexpected reaction of cochineal, iron sulfate, and cyanide, in which the original target was a type of red dye [9]. Through substituting the Fe species with other transition metals, such as Ni, Co, and Mn, plenty of Prussian blue analogs (PBA) have been identified. These compounds are also classified as a branch of the metal–organic framework (MOF) due to their varied metallic elements and 3D extending structures. The inexpensive cost of PBA is mainly due to the abundance of its constituent elements. Coupled with its straightforward preparation methods, PBA has been applied in various industries [17,18,19,20]. One of the most promising areas is electrocatalysis for water splitting. Compared with others, the controllable constituents and configurations render PBA unrivalled preponderances as an OER catalyst [21]. On the one hand, the Earth-abundant first-row transition-metal elements contained in PBA are often regarded as the most promising catalytic substances to replace the current commercially available noble-metal-based OER catalysts. On the other hand, the open-framework structure of PBA provides channels for mass transfer. Meanwhile, PBA can be readily converted to other materials with OER catalytic activity, such as transition-metal oxides, or to structures that could more effectively expose active sites, such as hollow structures [20,22]. All this means that the presence of PBA and its derivatives provides a cornerstone for the acquisition of novel green OER catalysts. There is no doubt that elaborate adjustments are needed to render PBA its catalytic potential, while the detailed mechanisms and principles are discussed below.

Based on the continuous efforts on characterization methods from numerous scientists, the cognitions of PBA are further intensified. Herein, with the in-depth understanding of the structure–property relationships, the updated design strategies for preparing PBA-based OER catalysts including the structural adjustment, synthetic technologies, and electrochemical measurements, as well as their advantages and disadvantages in electrocatalytic water splitting applications, are analyzed. Moreover, the introduction of irradiation into the electrolysis of water as part of driven energy for the reaction can be considered as an approach to accelerate the green catalysis, and the photoelectrolysis of water has similar reaction principle and process with the normal electrocatalytic water splitting. Therefore, applications of PBA in OER of photoelectrolysis of water are also reviewed to provide an overall outlook and hints of the challenges and opportunities in this research area.

## 2. Oxygen Evolution Reaction

### 2.1. Reaction Mechanisms

OER is an important semi-reaction in electrochemical devices mentioned above. Thus far, although scientists have devoted enormous efforts into the research of materials development, structural design, manufacturing techniques, characterization methods, and theoretical calculations, no catalyst has been discovered with combined considerably low overpotential, excellent stability, and ease of industrial production [23,24,25,26]. Hence, more in-depth understanding of the processes and mechanisms of OER is essential to address these issues.

Currently, the known pathway of OER is a four-electron process; Equation (1) shows the reaction under acidic condition, while Equation (2) represents the process in alkaline medium [27,28], where E^0^ represents standard electrode potential.

2H_2_O → O_2_ + 4H^+^ + 4e^−^ (E^0^ = +1.23 V) (1)

4OH^−^ → O_2_ + 2H_2_O + 4e^−^ (E^0^ = +0.40 V) (2)

The above equations illustrate that OER tends to proceed at positive potentials in acidic media, which greatly limits the discovery of available catalysts, and O_2_ prefers to evolve under alkaline conditions due to a lower redox potential. As previous studies exhibit, only iridium- and ruthenium-based catalysts have qualified performances under specific conditions [29].

After that, Man et al. further revealed the mechanism of electron transfer pathways by density functional theory calculation (DFT) [12]. Equations (3)–(6) demonstrate the reaction steps in acidic medium.

H_2_O + * ⇄ *OH + H^+^ + e^−^ (3)

*OH ⇄ *O + H^+^ + e^−^ (4)

*O + H_2_O ⇄ *OOH + H^+^ + e^−^ (5)

*OOH ⇄ O_2_ + H^+^ + e^−^ + * (6)

Equations (7)–(10) indicate the process under alkaline conditions.

OH^−^ + * ⇄ *OH + e^−^ (7)

*OH + OH^−^ ⇄ *O + H_2_O + e^−^ (8)

*O + OH^-^ ⇄ *OOH + e^−^ (9)

*OOH + OH^−^ ⇄ O_2_ + H_2_O + e^−^ + * (10)

where the asterisk (*) represents the active site during the reaction.

**Figure 2:**
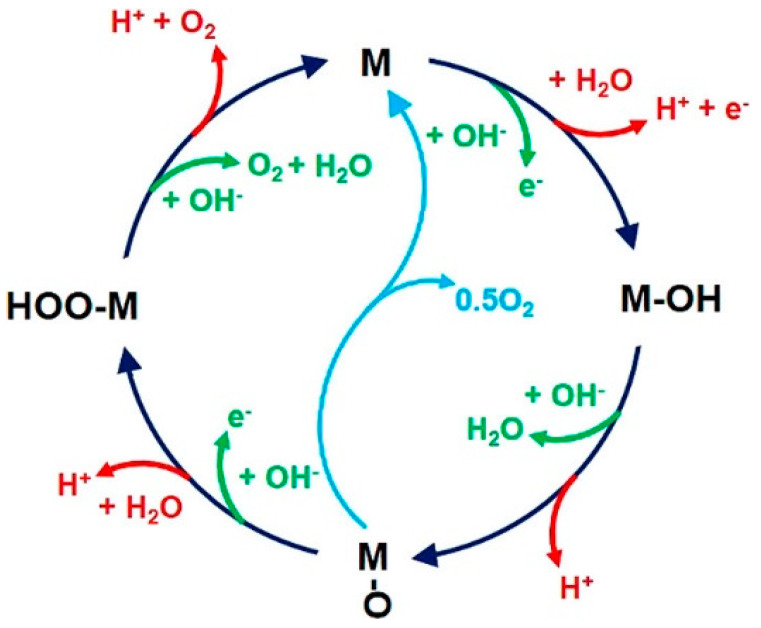
The cyclic diagram of OER. The red line (alkaline conditions) and green line (acidic conditions) indicate the products generated by each step when the OER takes place in different media. The dark blue line indicates the reaction process involving the formation of peroxide intermediate while the light blue represents the process by which oxygen is produced when two adjacent M-O intermediates react directly. Copied with permission from [31]. Copyright © 2018, American Chemical Society.

Although researchers still have different ideas concerning the exact formation mechanism of O-O bond [30], the above process is widely accepted. Another view expects that two adjacent *O intermediates can directly react to form O_2_, as shown in Figure 2 [31]. Besides, it is worth noting that the generation of by-product H_2_O_2_ is competitive with that of O_2_ under some conditions [27].

### 2.2. Design Principles and Evaluation Criteria for the Catalysts

In recent research, commercially available OER catalysts are still a series of noble-metal-based catalysts represented by IrO_2_ and RuO_2_ [16]. However, the high cost and scarce reserves of these precious metal-oxides make them challenging to work in sustainable industries. Simultaneously, these commercial catalysts typically require operating voltages of more than 1.8 V [24], which is considerably high. These factors together indicate the importance of developing highly effective catalysts.

For most electrocatalysts, the general design is aimed to reduce the electrical resistance, improve the catalytic activity, inhibit undesirable side-reactions, and maintain excellent stability under both electric and acid–base conditions [25,32,33,34]. More specifically, the process of OER usually occurs on the three-phase interfaces among the electrode, electrolyte, and evolving gas. Therefore, the catalysts are also expected to have rational structures to adequately expose their active sites and facilitate the rapid diffusion of electrolyte and gas. Based on such considerations, in addition to enhancing the activity and stability of the catalytic materials themselves, tactics such as improving the specific surface and adopting the porous structure in the design of the catalysts could be helpful in effectively exposing more active sites and raising the contact area between the electrolyte and electrode. Both strategies are discussed below.

To design more efficient catalysts and conduct horizontal comparisons between diverse materials, standardized parameters are expected to be established for the evaluation of their performances. There is no doubt that, besides the cost, two most indispensable indicators are the catalytic activity and stability. Researchers have thus proposed numerous testing methods and corresponding parameters to describe the capabilities of the catalysts in both aspects. Both overpotential (η) and Tafel slope have the priority to evaluate the overall activity of OER catalysts; while the former indicates the additional potential required to drive the electrochemical reactions over the equilibrium potential (1.23 V for water electrolysis), the latter is usually calculated by the current density, the actual number of charge transfers, and the overpotential [35,36]. These two indicators can usually be measured by cyclic voltammetry (CV) or linear sweep voltammetry (LSV) [37,38,39]. However, due to the difficulty of these two methods in surveying the polarization curve at steady state, some methods such as potentiometry or electrochemical impedance spectroscopy (EIS) have also been established [40]. The turnover frequency (TOF) is another parameter which measures the intrinsic activity of a catalyst, and it is defined as the normalized ratio between the generated gas and active sites in the electrode [41]. Otherwise, Faradaic efficiency (FE) has guiding significance since it is the ratio of experiment and theoretical values of the amount of resulted oxygen [42,43]. Moving forward to the stability issue, although the instability of a catalyst can be readily determined by chronoamperometry and chronopotentiometry, proving its stability usually requires additional assistance of CV cycling and LSV.

## 3. Prussian Blue Analogs

**Figure 3:**
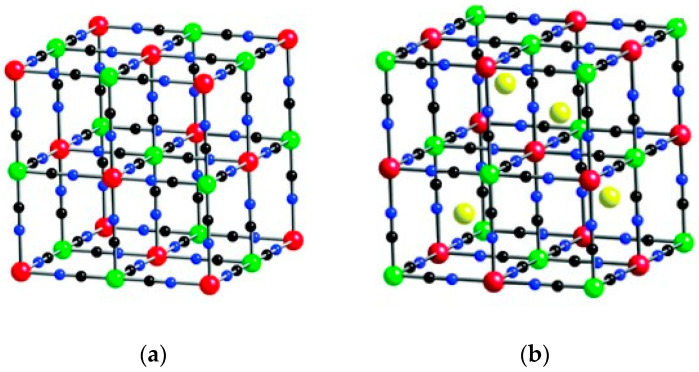
(**a**) The unit cell structure of PB in the possible Fm3m space group. The Fe^III^, Fe^II^, C, and N are represented by the red, green, black, and blue spheres, respectively. (**b**) An example crystal structure diagram of A_x_M_a_[M_b_(CN)_6_]. In this case, the A-sites metals are shown by the yellow balls. The M_a_ is demonstrated in red while M_b_ is in green, and the C (black) and N (blue) act as the bridge between them. The water molecules are not shown here due to the complexity of their orientation. Adapted with permission from [46]. Copyright © 2016, Royal Society of Chemistry.

Prussian Blue (PB), one of the most typical coordination polymers, was initially discovered in the beginning of eighteen century by a German pigment producer [44]. After that, scientists have concentrated on the investigations of PB and its analogs (PBA) by various means. Buser et al. issued the first report regarding the single crystal structure of Prussian blue [45]. They assumed that their synthesized PB (Fe_4_[Fe(CN)_6_]_3_·xH_2_O, x = 14~16) had the structure of face-centered cubic cells with the average lattice side length a = 10.166 Å, and that it belongs to Fm$\bar{3}$m or Pm$\bar{3}$m space group. Figure 3a demonstrates the unit cell structure of Prussian blue in the possible Fm$\bar{3}$m group [46]. The Fe^II^ and Fe^III^ are alternatively aligned on either side of the cyano-group, connecting each other by the coordination bonds. PB is a typical compound with mixed valence-state, in which Fe^II^ is surrounded by C and Fe^III^ is enclosed by octahedral N, thus jointly forming its backbone. According to the stoichiometry, to maintain the electronic neutrality of the whole compound, water molecules or vacancies are highly likely to exist in the framework. Further, researchers found that the water molecules can either coordinate to Fe^III^, which are part of the polymeric framework, or exist in the interstices [22]. These factors could affect the parameters of the unit cell, thereby influencing the whole electronic structure of PB [47].

Afterwards, researchers found that the Fe species could be substituted by other transition metal elements, which indicate the birth of PBA. PBA could be generally represented by the formula of A_x_M_a_[M_b_(CN)_6_]·nH_2_O, as shown in Figure 3b, where M_a_ and M_b_ indicate transition metals, such as Mn, Co, Ni, Cu, Zn, etc., and A_x_ indicates alkali or alkaline earth metal ions existing in the interstitial sites of the frame, such as Li, Na, Mg, K, etc., while the substitution does not have a devastating effect on the overall construction of the PBA [48]. In addition to being controlled by the diversity of its composition, the electronic properties of PBA can also be fine-tuned by the following methods such as electric field, temperature, radiation, chemical modification, etc. Due to the controllable preparation and variable compositions as well as their resulting alterations such as the electronic structures, PBA has been found to have extensive prospects in various applications, such as rechargeable batteries [49,50,51], supercapacitors [52,53], gas storage [54], photo/electro-catalysis [34], sensors [55], etc.

Regarding the OER catalysis, PBA has distinct advantages. On the one hand, the molecular-level tuning ability of the composition allows the PBA to have multiple catalytic transition metal atoms in a single polymeric materials framework, and these atoms are strongly coupled in the framework, making it easier for them to function synergistically. Meanwhile, this also grants PBA the ability to be further modified through chemical processes and transformed to other multi-metallic compounds [56], and the obtained materials are more likely to be endowed with enhancement in catalytic activity and stability. On the other hand, the controllable structure and size not only enables periodic arrangements of active catalytic sites but also helps PBA to act as the templates for more talented configurations. Moreover, the open framework of PBA could result in the acceleration of mass transfer during the reaction. These factors are the main reasons PBA is extensively applied in OER catalysis.

In this section, the recent progress of the methodologies regarding the fabrication and characterization techniques of PBA is reviewed to provide a more in-depth understanding of its structure–property relationships. Further, the updated design strategies for PBA to be applied in OER catalysis are analyzed. The discussion is roughly divided into two parts: (1) modifying PBA by doping, chemical decoration, or setting it into distinctive structures while maintaining the basic structural unit of PBA; and (2) using PBA as a precursor to prepare other compounds, such as transition-metal oxide, or unique configurations, such as porous structure, in which at least one of the composition or construction of PBA is destroyed. Additionally, according to the similarity of reaction mechanisms of electrolysis and photoelectrolysis of water, the advantages and disadvantages of PBA applied in the latter field arediscussed as well.

### 3.1. Methodologies for PBA Synthesis and Characterization

**Figure 4:**
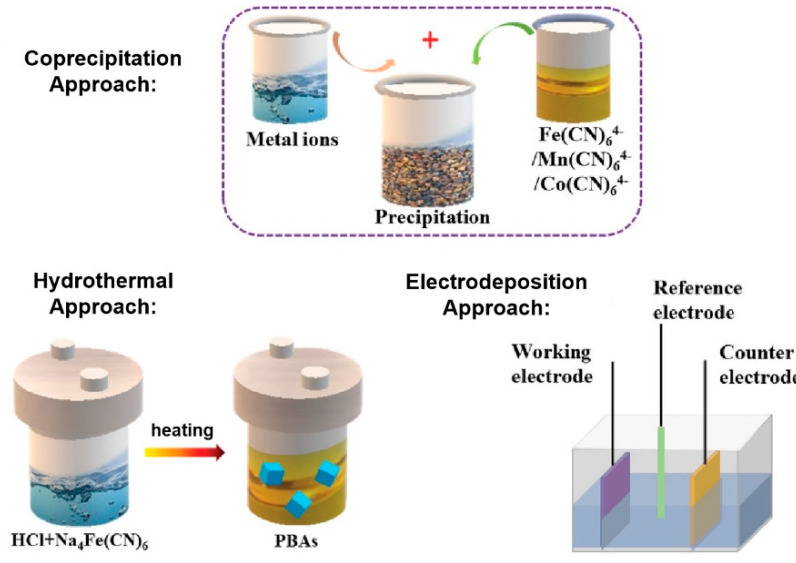
Schematic diagram of the preparation processes of co-precipitation, hydrothermal, and electrodeposition approaches. Adapted with permission from [9]. Copyright © 2019 WILEY.

In general, the PBA with regular morphology and single crystal is always desired for various applications. The choice of different synthetic methods plays a decisive role in the crystallography of the PBA products, and thus determines the property and application of PBA. Various synthetic routes have been established, such as co-precipitation, hydrothermal, electrodeposition, microemulsion, microwave method, etc., and the first three are more popular for PBA of electrochemical applications [9,57], as demonstrated in Figure 4.

Many scientists favor the co-precipitation approach because of its simple operations and low cost. For example, Wessells et al. successfully synthesized homogeneous copper hexacyanoferrate (Cu-HCF) and Ni-HCF particles by this method [58]. The former is obtained by reacting Cu(NO_3_)_2_ with K_3_[Fe(CN)_6_] at room temperature, while the latter has similar reaction conditions with additional heating to 70 °C. This approach is assumed to have great potential for scaling-up. However, the irreversibility of this route results in the loss of control during the reaction process. Hence, it is necessary to add some external assistance, such as coordination agents, to slow down the crystallization process. Ming et al. introduced polyvinylpyrrolidone (PVP) as the assistant in the preparation process of PB. They found that the alteration of PVP content and pH value can jointly influence the size, surface roughness, shape, and particle size distribution of PB crystal [59]. Zakaria et al. added trisodium citrate as a chelating agent to their reaction system. With the increase of the chelating agent, the strengthening of crystallinity was proved by the experimental results of SEM, XRD, and NMR [60]. These findings suggest that the crystallization process of PBA could be interfered by chelating agents to form regular crystallites.

The hydrothermal method is another broadly used strategy, and it has better adjustment ability to tune the crystal shape and size than the former co-precipitation way. The morphology of PBA produced by this route is usually determined by the structure of its precursors. Liu et al. found that the acid content (in this case, hydrogen chloride (HCl)) can significantly affect the morphology of the fabricated PB, while the added HCl can also prompt the formation of PB [61]. In addition, the reaction temperature and surfactant are also pivotal factors to influence the lattice parameters. They thereby obtained the PB with regular cubic structure, concave cubic structure, and spherical structure according to the alteration of reaction conditions.

The electrodeposition method of PB synthesis was initially proposed by Neff [62], and could in-situ generate PBA on various substrates. The morphology of the as-prepared PBA thereby is strongly affected by the substrate. Simultaneously, this method is also applicable for materials that require further modification or reaction of PBA. Isfahani et al. prepared a series of PB films with different preparation time on the indium tin oxide coated glass (ITO) substrate with a fixed applied potential [63]. By FESEM, EDX, and CV characterizations, they found that the deposition time significantly changed the uniformity of the PB surface as well as the stability. Longer deposition time leads to rougher surfaces of the PB films, and the corresponding improvement of the catalytic activity resulted from the exposure of more active sites caused by such morphologies.

Characterizing the chemical composition, morphology, and crystal structure of PBA with the maximum feasibility is the basis for the accurate understandings of the structure–property relationship. Currently, the commonly used testing methods for the above information include scanning electron microscopy (SEM), transmission electron microscopy (TEM), X-ray diffraction (XRD), X-ray photoelectron spectroscopy (XPS), X-ray absorption spectrum (XAS), neutron diffraction (ND), etc. [20,46]. In addition, re-characterizing the catalyst after the reaction can determine the changes in stoichiometry, the valence state of the elements, and the crystal structure, and these factors could indicate the exact active sites, the stability of the catalyst, and various other information. The nanostructures of most solid samples could be characterized by SEM and TEM. To further improve the signal-noise ratio, cryogenic electron microscopy technique (cryo-EM) can be utilized. The extremely high resolution of cryo-EM enables its promising ability to provide molecular-scale evidence for the kinetics of the reaction [64]. The chemical constituents of the catalyst and the corresponding valence state can be determined by XPS, while XAS usually measures the electron structure and the coordination environment at the atomic level. By comparing the XPS and XAS of the samples before and after the OER reaction, Zhao et al. found that Co-Fe oxides and hydroxides were in situ generated on their Co-Fe-S@PB nanoboxes during the reaction process, thus narrowing the range of possible reaction pathways [65]. Besides, the crystal structure, including defects such as vacancies, is crucial for PBA as a catalyst. On the one hand, the crystal type directly determines the electronic structure of PBA. On the other hand, the presence of vacancy could identify the difficulty of mass transport and the generation of grain boundaries, thus affecting the contact between the active site and the reactant. In addition to the conventional testing methods regarding the crystallography, such as powder XRD, other novel strategies have been proposed and still need developments [66]. Simonov et al. determined a variety of non-random vacancy arrangements in PBA by X-ray diffusion scattering with the assistance of three-dimensional difference pair distribution function analysis and Monte Carlo simulations [67]. These vacancies were difficult to detect by traditional methods. The microporous characteristics and the vacancy-network polymorphs they observed explained the electrochemical performances obtained in previous experiments. These results act as the foundation of more exact cognitions for the structure–property relationships of PBA, especially concerning the storage and transport, as well as the applications of vacancy engineering.

### 3.2. Applications

**Figure 5:**
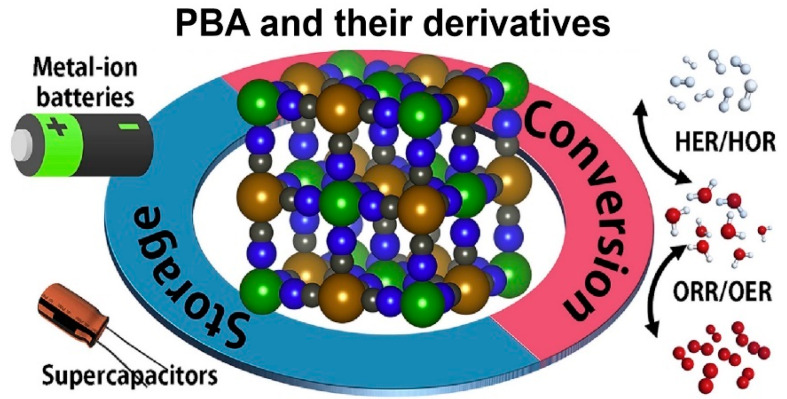
Primary applications of PBA in the field of energy storage and conversion. Adapted with the permission from [68]. Copyright © 2020 Elsevier.

According to the continuous enthusiasm from researchers for the synthesis, characterization and many other aspects of PBA, the fields of its application are also expanded continuously, as demonstrated in Figure 5 [68]. The diversity of its components and the peculiarity of its structure result in the large potential of PBA to be applied in OER. The periodic open-framework structure enables PBA to be anticipated to have higher specific surface areas than other catalysts, which means that it could conveniently expose the active sites and facilitate the diffusion of substances. Its controllable chemical compositions allow it to have adjustable active components and modifiable surfaces. However, its high charge resistivity is still the core barrier limits its possibilities in electrochemistry. Pristine PBA typically requires at least 340 mV onset potential and the Tafel slope are approximately 80 mV dec^−1^ [69]. At present, two widely used strategies for applying PBA in OER include modifying and using it as a precursor to construct other functional electrocatalysts. Following these guidelines, recent progress in the synthesis, structures, morphologies, and electrocatalytic properties for PBA-based OER catalysts are summarized here.

#### 3.2.1. OER in Electrolysis of Water

##### Modifying PBA

As one of the most classical coordination polymers, PBA has the general advantages of this category of materials, such as high crystallinity, large specific surface area, and strong metal–ligand interactions. Although these features are likely to render PBA to be suitable as a type of heterogeneous catalyst, it also brings some problems. According to the crystal field theory, in the 3D networks of PBA, the metal atoms coordinated with C show low-spin state contributed by the d-$\pi^{*}$ back-donation, while on the opposite side of the cyanide groups, the d-orbitals of N-coordinate metal atoms become unstable because of the σ-donating and thereby exhibit high-spin state. Moreover, due to the existence of interstitial sites and vacancies caused by the deficiency of cyanometallates, water molecules are very likely to exist in the crystal structure of PBA. Together with the interstitial alkali metal ions, these factors jointly determine the electronic structure of PBA and thereby affect its redox performances [22,70]. Based on these physicochemical properties, PBA generally are not able to afford satisfactory current densities. Besides, although it performs well under alkaline conditions, its catalytic properties in neutral and acidic medium have rarely been reported.

Thus far, various strategies have been developed to enhance the application of PBA in OER. Scientists chose to construct doped PBA with electrons conductive nanoparticles or directly growing PBA on electron-conductive substrates. Composite materials formed by directly combining the PBA and conductive substances are likely to possess the advantages of both components [71]. Nevertheless, how to distribute the ingredients evenly remains as an unsolved problem. Graphene was initially considered because it has almost no charge resistance in theory. In practical applications, however, both graphene oxide (GO) and reduced graphene oxide (rGO) as the derivatives of graphene tend to have higher priorities due to their cheaper price. Ghasemi et al. prepared GO/nickel-iron hexacyanoferrate (Ni-Fe-HCF) nanocomposites and found that they had relatively fast charge transfer rates and excellent stability under acidic conditions [72]. Although their work did not illustrate the performances of such materials in OER, it set stairs for further explorations.

**Figure 6:**
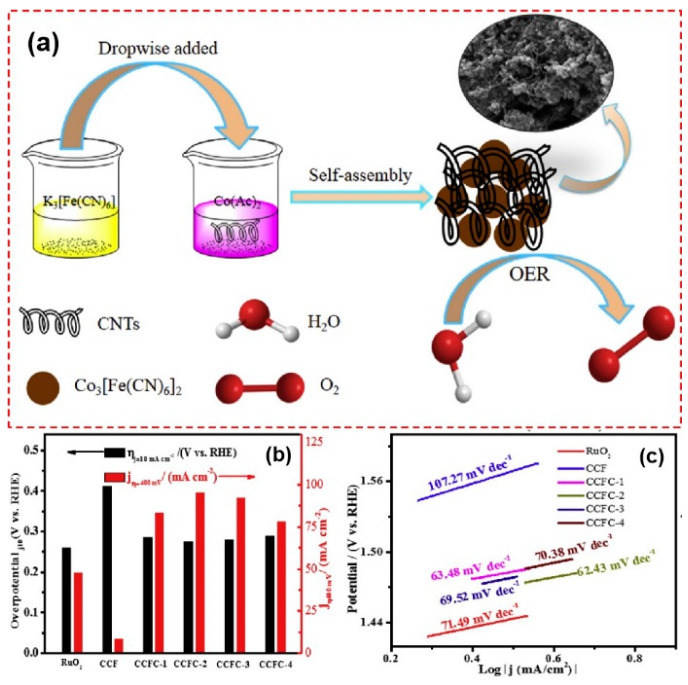
(**a**) Schematic diagram of the reaction processes of Co-HCF/CNT nanocomposites; (**b**) comparison of the overpotential for reaching 10 mA cm^−2^ (black pillars) and current densities at an overpotential of 400 mV (red pillars) of RuO_2_, pure Co-HCF (CCF), Co-HCF with 0.1 g/L CNT (CCF-1), Co-HCF with 0.2 g/L CNT (CCF-2), Co-HCF with 0.3 g/L CNT (CCF-4), and Co-HCF with 0.4 g/L CNT (CCF-4); and (**c**) Tafel plots of RuO_2_, CO-HCF and Co-HCF/CNT nanocomposites with different loading. Adapted with the permission from [74]. Copyright © 2019 Elsevier.

After that, Ramos et al. used rGO/copper oxide nanocomposite as the precursor to synthesize rGO/copper hexacyanoferrate (Cu-HCF) nanocomposite films by electrodeposition assisted by KCl and K_3_[Fe(CN)_6_] in aqueous solutions [73]. Through XRD and Raman mapping, the homogeneities of the films are confirmed. They also indicated the catalytic potential of the nanocomposites through CV test. Compared with graphene, one-dimensional reinforced materials may more easily form a conductive network in the bulk material. Zhang et al. used co-precipitation as their preparation method for cobalt hexacyanoferrate (Co-HCF)/carbon nanotubes (CNT) nanocomposites [74], as shown in Figure 6. The as-formed spherical structures theoretically increased the specific surface area. Smaller Tafel slope (62.43 mV dec^−1^) and overpotential (274 mV at 10 mA cm^−2^), which are apparently lower than those of previously reported Co-HCF, proved the synergistic effect of these constituents. Using the same raw materials, Husmann et al. generated the interface of water and 1,2-dichlorobenzene where the PBA is electrodeposited on free-standing CNT films under the control of pH and external voltage [75]. The operation at the immiscible solution interface may facilitate the uniform assembly of these two materials so that the resulting film is more likely to have homogeneous electrochemical activity.

Chemical modifications are compromised with composition adjustment and surface engineering. The main reason reinforcements could lead to the former is that the presence of metal atoms with different oxidation states in PBA may result in different microstructure and crystal morphology. Tang et al. reported a nickel-based PBA (Ni-PBA), whose overpotential (30 mV at 5 mA mF^−1^) and Tafel slope (41 mV dec^−1^) are considerably lower than that of pristine PBA [69]. They inserted sodium ions to alter the oxidation of Ni, while the electrochemical cycling of sodium ions brought the defects and disorders. Similarly, Han et al. reported a series of Fe_x_Co_2-x_[Fe(CN)_6_], which could work in a wide pH range (pH 0–13) [70]. They partly replaced the electrocatalytic center Co by doping Fe, thus forming the Fe^II^-CN-Fe^III^ pairs. The additional Fe has been proved to enhance the electron transfer in the framework, thus higher current densities (peaking at over 100 mA/cm^2^) could be achieved. Compared with doping electronic conductive nanoparticles, this could be a more straightforward approach. The broader application of surface engineering can be attributed to the better interfacial matching between the electrode surface and the catalyst. Han et al. designed a novel approach for the preparation of Co-PBA films [76]. They first grew CoO_x_ (O_x_ stands for anions that can compete with cobalt ions, such as CO_3_^2−^, OH^−^, and O^2−^) on fluoro-doped tin oxide (FTO) to fabricate the cobalt nanowire array, and the [Fe(CN)_6_]^3−^ cubes were grafted onto the nanowire through the chemical etching process. Their films have remarkable reinforcements in not only catalytic performances (smaller overpotential required when pH = 2) but also both long- and short-term stabilities (1 ≤ pH ≤ 13). Aksoy et al. deposited amorphous cobalt pentacyanoferrate/poly(4-vinylpyridine) (Co-PBA/PVP) on an FTO electrode to increase the active cobalt sites [77]. Although grafting PVP did not significantly improve the intrinsic activity (262 mV overpotential for [CoFe(CN)_6_@FTO] and 284 mV overpotential for [CoFe(CN)_6_-PVP@FTO] when reaching 2.6 × 10^−3^ s^−1^ of TOF), which could be attributed to the similar structural units and chemical environment of the cobalt and much lower overpotential (510 mV) needed to achieve 1 mA cm^−2^ of current, while the value for [CoFe(CN)_6_@FTO] to achieve the same current density is higher than 600 mV.

Additionally, unique structures, especially low-dimensional structures, can not only alter the overall electronic structure of the material but also increase the specific surface area, thereby promoting the contact between electrolyte and electrode to achieve optimum exposure of active sites. Moreover, approaches of utilizing defect chemistry such as vacancy engineering can also significantly tune the band structure, conductivity, catalysis, as well as the overall structure of PBA [78]. Bui et al. developed a facile route based on ion exchange [79]. The potassium ferricyanide reacted with vertically-oriented 1D nanoarray cobalt hydroxycarbonate thin film in aqueous solution, and in-situ deposited Co_3_[Fe(CN)_6_]_2_. Through morphology investigations, they found that the target material could be constructed without damaging the 1D nanowire structure and orientation, which is assumed to be the main reason for the enhancement of directional electron transportation. Further electrochemical experiments confirmed this assumption, the obtained Co_3_[Fe(CN)_6_]_2_ film exhibited very close value with commercial Ir(20 wt%)/C catalyst of the required potential when reaching 10 mA cm^−2^ current density, which was +1.65 and +1.63 V, respectively. The same group further perfected this route by using electrochemical deposition to promote the aqueous reaction, and they successfully gained high-performance hexacyanoferrate films for overall water electrolysis [80]. Xiao et al. designed the multi-level nanostructure by mixing the PBA and MOF with various arrangements through in-situ ion exchange [81]. The as-formed nanostructures enabled active sites to be exposed and electrons to be conducted more quickly, and these features could be further improved by the synergistic effect. At the same time, the strong electronegativity of cyano groups helps the nickel, which is the catalytic activity center, to lose electrons, and thereby enhance its catalytic activity directly. The calculated equilibrium constant proves the excellent catalytic performance of the structure.

**Figure 7:**
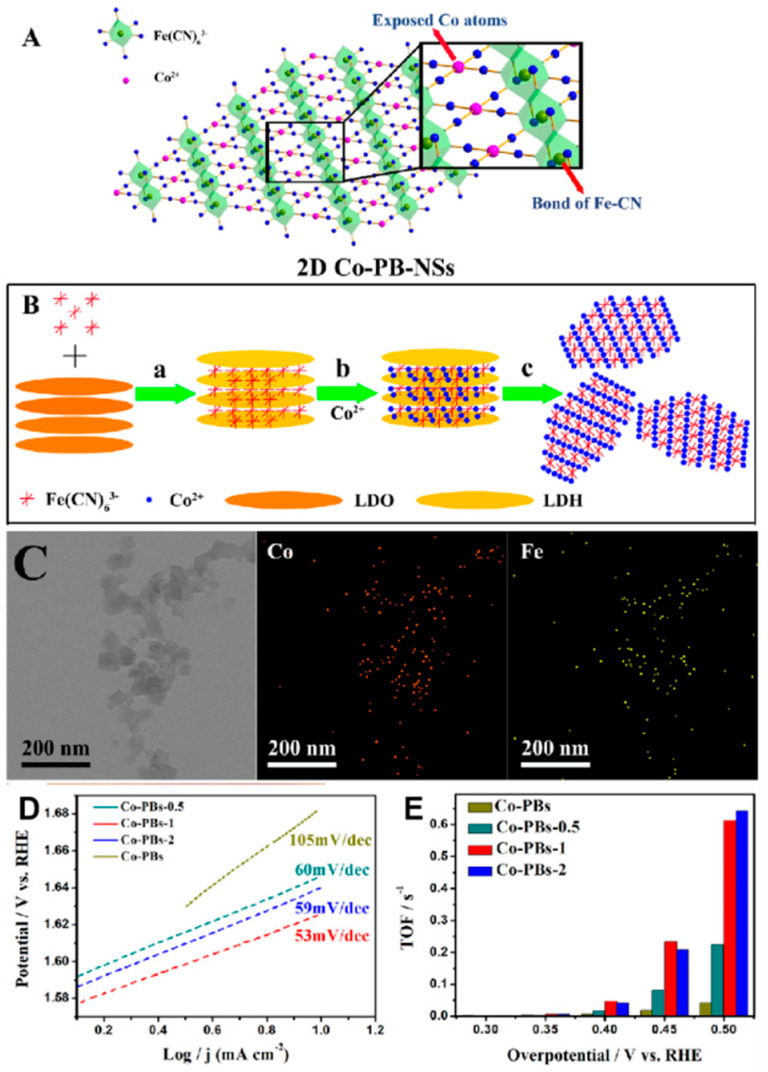
(**A**,**B**) The schematic diagrams of the 2D layered CoFe-PBA nanosheet and its synthesis process; (**C**) STEM-EDS elemental mapping images of Co and Fe elements, respectively, show their distributions in the fabricated nanosheets; (**D**) Tafel plots of the CoFe-PBA nanosheets formed with different loading of the conductive substrates; and (**E**) TOF/oxidative cobalt site (Co^II^). Adapted with the permission from [82]. Copyright © 2020 Elsevier.

Huang et al. dispersed and complexed Fe(CN)_6_^3−^ and Co^2+^ in the interlayer of the layered template, forming 2D laminar CoFe-PBA ultrathin nanosheets with the thickness around 1.71 nm [82]. The schematic diagram of the nanosheets and their fabrication process are shown in Figure 7A,B. Within the obtained nanosheets, it could be observed from the STEM-EDX elemental mapping that the Fe and Co are uniformly distributed, suggesting the positive effect of the fabrication methods, as demonstrated by Figure 7C. Benefiting from the characteristic 2D structure, Co-PBA nanosheets gained higher electron mobility and active site exposure. To further improve the conductivity of the nanosheets, the authors added conductive substrates into their samples. In Figure 7D,E, the electrochemical performances of the as-formed materials are measured, and the number after Co-PBs represent different loading of the conductive substrate. Both overpotential and Tafel slope were obtained with smaller values, which was 448 mV at 10 mA cm^−2^ and 105 mV dec^−1^, respectively, indicating that faster kinetics was achieved. Yu et al. introduced a series of cyano group vacancies by bombarding Ni-Fe-PBA with ionized nitrogen [78]. Changes in the local electronic structure and coordination environment of PBA inhibited Fe leaching during the OER reaction, which makes it easier to self-rebuild the active layer of Ni-Fe oxides. Therefore, better catalytic activities were obtained (overpotential of 283 mV at 10 mA cm^−2^ and Tafel slope of 54 mV dec^−1^).

In general, when PBA is applied directly to OER catalysis, its high charge resistivity remains the most difficult obstacle to overcome. Hence, researchers are supposed to propose more ways to overcome this problem. At the same time, the unparalleled excellent arrangement of catalytic substances and remarkable stability of PBA, especially in acidic conditions, are also likely to be referenced in more OER catalysts.

##### Using PBA as the Precursor

The as-mentioned advantages of PBA are assumed to boost its performances to be used as a precursor for various OER catalysts. On the one hand, PBA contains both metal components and cyano-bridges. Generally, the periodic arrangement of PBA could prompt a homogeneous distribution of the active atoms. Simultaneously, due to the variety of its constituents, PBA has the inherent superiority of being converted into different metallic compounds. Traditional Earth-abundant catalysts for OER are mainly transition-metal oxides and hydroxides. Since the activity of transition metals is dependent on their vacant d-orbitals and unpaired electrons, which are significantly affected by nearby coordinating groups, more metallic compounds have been discovered and become competitive in recent years, such as phosphates, borates, selenides, and halides. The controllability of metal ions with diverse valence states makes PBA to have incomparable potential to be converted into the above inorganic functional materials. Moreover, since M_a_ and M_b_ are mostly unidentical metals, PBA provides a more natural way to prepare multi-metallic compounds.

On the other hand, the structure versatility of PBA, particularly its flexible topology and nanoscale size, is of great necessity for excellent OER catalysts. Within PBA crystals, in addition to the absence of M-site metal ions or cyano-groups, water molecules may exist in these vacancies or interstitial positions. At the same time, varieties of alkali metals could be intercalated or deintercalated. These factors are non-negligible for the electronic property and crystal morphology of PBA. Meanwhile, with the development of characterization techniques, more evidence shows that different structures of compounds of the same element often have different properties. Hence, PBA provides a stepping-stone for many talented structures [83].

Depositing the PBA onto conductive substrates is one of the most widely used approaches because PBA not only can be directly converted into highly active catalysts but also provides strong connections for a variety of substances, and the substrates can work as the continuous frameworks for the hybrids. Nickel foam (NF), as one of the most extensively applied substrate, meets the above conditions while it can also serve as the source of nickel, which is considered to have fabulous OER catalytic activity and affordable price [84,85,86].

**Figure 8:**
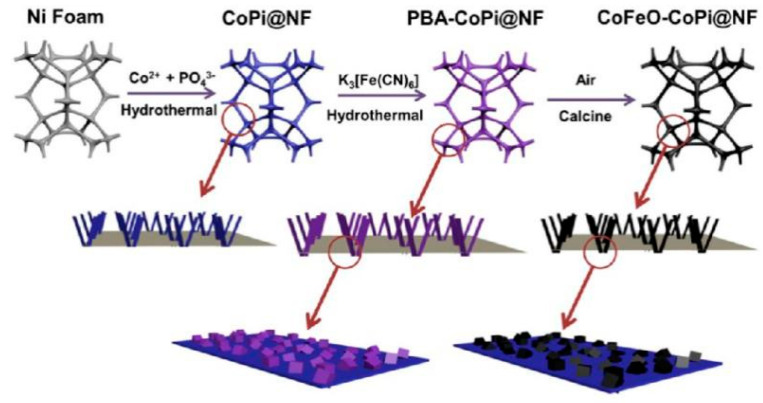
Schematic illustration of CoFeO-CoPi@NF. Adapted with permission from [87]. Copyright © 2020, American Chemical Society.

Guo et al. designed a facile route to prepare the CoFeO-CoPi@NF integrate catalyst [87], and the schematic diagram is shown in Figure 8. They deposited cobalt phosphate (CoPi) on the (NF), and then grafted CoFe_2_O_4_ onto CoPi, which was produced from the CoFe-PBA by calcination in air. The reconstruction of the surface between CoFe_2_O_4_ and CoPi induced electrons to transfer from the former to the latter. This directly renders the cobalt species in the former to have more electron traps, thus it is easier to attract the charges lost during reduction reaction, indicating higher OER activity. Their work evidenced that strong chemical coupling between molecules could be constructed with the aid of the intermediate PBA, resulting in the d-band center moving toward a lower level. Yuan et al. used the same substrate to provide a 3D framework, and their precursor Ni-Co-PBA nanocubes were evenly germinated on the NF [88]. Through the ion exchange process, their final product Ni_3_S_2_@MIL-53(NiFeCo) was formed and proved to have the 1D nanowire structure, which was uniformly distributed on the NF. The rational synthesis technique and structural design are considered as the cornerstone for the conspicuous enhancements in electrocatalytic performances (required 249 mV to achieve 100 mA cm^−2^ and the Tafel slope was 14.8 mV dec^−1^ in 1 M KOH). What is more, although carbon paper (CP) itself does not have catalytic activity, its perfect charge conductivity and porous structure makes it a broadly used substrate for various catalysts [89]. Ishizaki et al. proposed a novel route to distribute Co-Fe-PBA evenly and compactly on CP by surface modification, and then calcinating in air makes PBA pyrolysis into Co-Fe oxide, which has higher catalytic activity [90]. By altering the composition, the lowest overpotential value of 0.46 V vs. RHE at 10 mA cm^−2^ was obtained, demonstrating apparent reinforcements toward OER catalysis.

**Figure 9:**
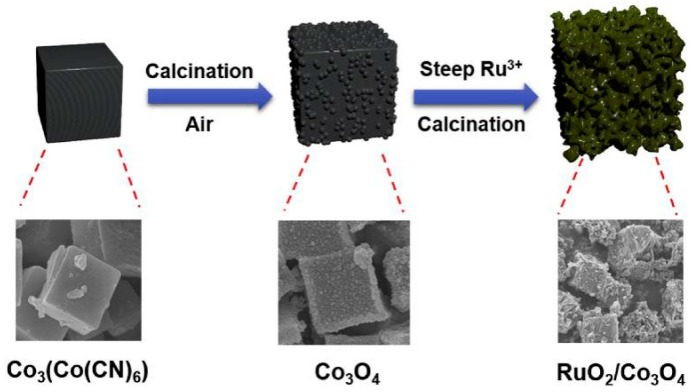
Schematic illustration of RuO_2_/Co_3_O_4_. Copied with permission from [91]. Copyright © 2020, Elsevier.

More catalysts tend to inherit the structural features of PBA. Within the PBA, the coordination bonds between the M-site metals and the cyano-groups are usually not strong enough to endure external conditions such as elevated temperature or guest ions. Thus, the coordination bonds are prone to be destroyed while maintaining the framework [22]. Guo et al. prepared RuO_2_/Co_3_O_4_ hybrid nanocubes through calcinating and ionic impregnating the Co_3_[Co(CN)_6_] [91], as shown in Figure 9. SEM element mapping and EDX together demonstrated the excellent dispersion of RuO_2_ on Co_3_O_4_. The pores created by calcinating led to more active sites of RuO_2_ being exposed, and the synergetic effects of the two metal atoms accelerated the electron transfer. Together, these factors determined that, although there was only 0.26 wt% of ruthenium, the hybrid material achieved unprecedented catalytic activity in comparison with all raw materials (required overpotential of 302 mV to achieve 10 mA cm^−2^, Tafel slope was 74.37 mV dec^−1^). Through a similar process, Ahn et al. transformed Co-PBA into single-phase CoS_2_ with well-dispersed ~4 nm pores by gaseous vulcanization [92]. The obtained electrode had improved overpotential (298 mV to reach 10 mA cm^−2^) and Tafel slope (94 mV dec^−1^) performance. A much smaller Tafel slope was achieved with the Mn_1.2_Fe_0.8_O_3_ prepared by Ma et al., which was fabricated through annealing Mn_3_[Fe(CN)_6_]_2_·nH_2_O [93]. The cubic bixbyite only required overpotential of 245 mV to gain 10 mA cm^−2^, and the Tafel slope was 38 mV dec^−1^.

**Figure 10:**
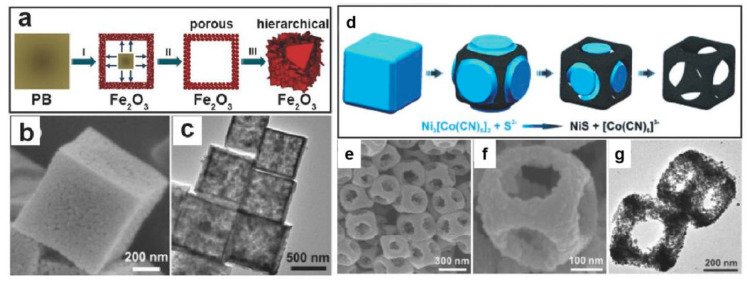
(**a**) Schematic diagram of the fabrication process of Fe_2_O_3_ nanoboxes from PB (Step Ⅰ: the formation of Fe_2_O_3_ from PB under a large temperature gradient below 350 °C along the radical direction; Step Ⅱ: Annealing at 550 °C; Step Ⅲ: the transformation of porous shell structure into hierarchically structure shell consisted of Fe_2_O_3_ nanosheets.); (**b**) FESEM images of the Fe_2_O_3_ nanoboxes; (**c**) TEM of the Fe_2_O_3_ nanoboxes; (**d**) schematic diagram of the fabrication process of NiS nanoframes from NiCo-PBA; (**e**,**f**) FESEM of the NiS nanocages; and (**g**) TEM of the NiS nanocages. (**a**–**c**) Copied with permission from [94]. Copyright © 2012, American Chemical Society; (**d**–**g**) Copied with the permission from [95]. Copyright © 2015 WILEY.

Based on the architecture of PBA, numerous structures could be further constructed. One of the most noteworthy architecture is the hollow structure, which is usually the combination of functional shell and internal pores or channels. Compared with solid constructions, such arrangements can significantly reduce the overall mass while maintaining the loading density of active sites. At the same time, the added cavities help more effortless mass transfer within the catalyst, which leads to closer contact between the active sites and the reactants. Two examples of the hollow structure are demonstrated in Figure 10 [94,95].

**Figure 11:**
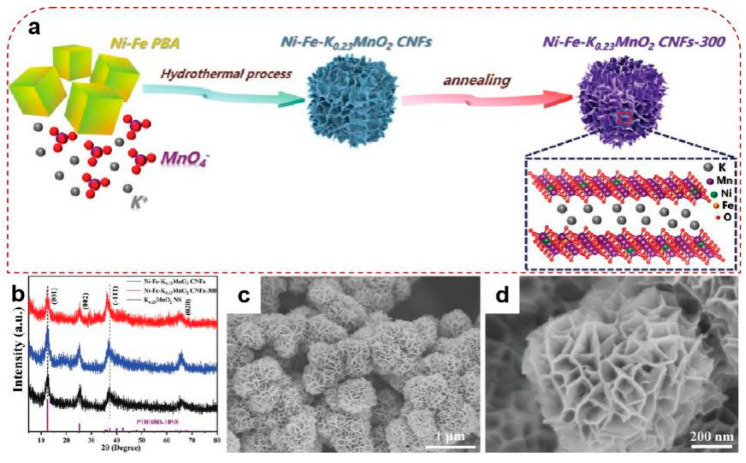
(**a**) Schematic diagram of the fabrication process for Ni-Fe-K_0.23_MnO_2_ nanoflowers (Firstly, the Ni-Fe-PBA nanocubes were fabricated through the co-precipitation approach. Then the Ni-Fe-K_0.23_MnO_2_ CNFs are formed by a hydrothermal reaction with the assistance of heating to 180 °C for 2 h. The further annealing process at 300 °C for 2 h in Ar atmosphere were aim to gain Ni-Fe-K_0.23_MnO_2_ CNFs-300.); (**b**) XRD of the nanoflowers compared with the raw material K_0.23_MnO_2_ nanosheets; and (**c**,**d**) FESEM images of the nanoflowers. Adapted with permission from [99]. Copyright © 2020 WILEY.

Zhao et al. synthesized CoFeS_x_@Co_3_[Fe(CN)_6_]_2_ with the closed single-shell hollow structure [65]. Their raw material was the CoFe-PBA nanocubes, which acted as the templates. After that, XPS and XAS demonstrated that the Co-S@PB and Co-Fe-S@PB nanobox heterostructures were generated through sulfidation. The electrocatalytic reinforcements were proved by smaller overpotential (286 mV at 10 mA cm^−2^) and Tafel slope (37.84 mV dec^−1^), as well as higher TOF than raw materials (1.03 s^−1^ at the overpotential of 300 mV). These enhancements could attribute to both the entire access of reactants to the active sites and the rapid departure of produced gas, which were induced by the advanced construction. Moreover, their products have also been found to possess remarkable stability across all pH range. Huang et al. gained similar conclusions regarding this structure [96]. Their Co-Fe-P nanoboxes were synthesized by the one-step co-precipitation reaction using Co-Fe-PBA as the template. They found that the supersaturation of the solution had a positive effect on the directional dissolution–recrystallization process, which promoted the formation of the hollow structure. Extraordinary OER catalytic performances of this nanobox was gained, with the lower overpotential of 235 mV to reach 10 mA cm^−2^ and Tafel slope of 34 mV dec^−1^. The open hollow structures require more open channels or pores on the surface of nanobox; in other words, they could be termed as nanocages. This construction allows the reactant to acquire easier accessibility to both the inside and outside active sites of the particle, thus increasing catalytic efficiency. Nevertheless, because of the complexity of their frames, their synthesis is often harder than that of nanoboxes. Xie et al. doped the Co_3_[Fe^III^(CN)_6_]_2_ with extra Fe to form Co_α_Fe^II^_β_Fe^III^[Fe^III^(CN)_6_]_σ_. Then, the Fe-CoP nanocages were produced through a low-temperature phosphating process [97]. The improvement in catalytic activity can be ascribed to the holes on the surface of the nanocubes that can be clearly observed in SEM and TEM, which granted the accessibility of the reactants to contact with inner active sites more easily. Kahnamouei et al. designed a one-step route to fabricate the CoFe nanocages. Then, the Ni-Co-S nanosheets were uniformly electrochemical deposited on the nanocages to further enhance the catalytic property of the hybrid [98]. Besides, PBA can also be used as a sacrificial template to synthesize many other capable configurations. Liao et al. designed the Ni-Fe-K_0.23_MnO_2_ cubic nanoflowers [99]. The starting material Ni-Fe-PBA acted as the template, and then the cubic nanoflowers were made through the in-situ chemical etching under hydrothermal conditions, as demonstrated in Figure 11. FESEM, TEM, and XRD together demonstrated that the etching process, as shown by Equation (11) [99], caused the Ni and Fe doped K_0.23_MnO_2_ nanosheets forming in the morphology of nanoflowers with the assistance of PBA nanocubes. While the concentrated Mn^3+^ resulted in the production of oxygen vacancies, which further led to the distortions, dislocations, and cavities. Such constructions theoretically maximize the exposure of the active sites, while introducing Fe and Ni ions into the lattice of MnO_2_ alters its inherent energy band level and electronic structure, resulting in a smaller charge resistivity. The nanoflowers thereby gained better electrocatalytic performances than current reported Mn-based and noble-metal-based OER catalysts, which could be proved by the lower overpotential (270 mV at 10 mA cm^−2^) and smaller Tafel slope (42.3 mV dec^−1^).

K^+^ + MnO_4_^−^ + KNi[Fe(CN)_6_] → Ni-Fe-K_0.23_MnO_2_ + CO_3_^2−^ + N_2_ (11)

Chen et al. proposed fabrication of virus-like Co-N-C nanoparticles, which was gained through carbonization of the template Co_3_[Co(CN)_6_]_2_ [100]. Within the nanoparticles, the Co-N-C acts as the core and the carbon nanotubes were dispersed vertically and uniformly on the outer surface. The rough morphology and synergies of Co with N resulted in its superior OER catalytic properties and good alkali resistance. Moreover, its impressive mechanical properties made it a promising candidate for flexible electrochemical devices.

All things considered, PBA could be classified as an excellent precursor for various OER catalysts. On the one hand, PBA could simplify the preparation routes of both transition metal-based and metal-free OER catalysts while maintaining their catalytic activity. It also promises the alliance of multiple catalytic substances becomes possible. On the other hand, the structural characteristics of PBA enable a great deal of further improvement to many talented configurations. More importantly, PBA is expected to become the focus for the combination of these enhanced catalytic substances and constructions.

#### 3.2.2. OER in Photoelectrolysis of Water

At present, the investigation of electrolysis of water is more comprehensive than before. The most important significance for electrolysis of water is the conversion of electricity from other sources of clean energy, such as wind, tidal, and light, into the form of chemical bonds. After that, burning these chemical fuels is the key to realize the circulation of energy. Among them, solar energy has been widely developed since it is more adequate and stable than other sources.

For the use of solar energy, transforming the light into electricity through solar cells is one of the most successful directions. However, the complicated electrical energy storage and unstable grid-connected voltage problems hinder the large-scale application of the solar cells in daily life. In contrast, solar fuels, which can transform solar energy into chemical energy stored in generated compounds, have received progressive attention. In the context, photoelectrochemical (PEC) water splitting process is one of the promising methods to generate a green energy source—H_2_ via solar energy. The discovery of PEC water splitting can be traced back to 1972: Fujishima and Honda firstly reported an experiment with using monocrystalline TiO_2_ crystals as photoanode to perform light-driven water splitting reactions without applying electrical bias [101]. This report opened up a brand-new solar energy conversion method, stimulating scientists to explore new PEC electrode materials with better performance. Up to now, the research on electrode materials for solar water electrolysis is mainly focused on semiconductors with wide band gaps [102,103,104,105]. Several metal oxides, such as Fe_2_O_3_, WO_3_, and BiVO_4_, are the ideal semiconductors for PEC water splitting electrode due to their rich storage, the capability in working under harsh environment, and matched band gaps for the high voltage (1.6–1.7 V) demand of water splitting reaction [106]. In recent research, Pihosh et al. published a report by using BiVO_4_ as photoanode to connect with double junction GaAs/InGaAsP device to enhance its energy transform efficiency, and achieve a new record of 8.1% solar-to-hydrogen energy efficiency [107]. This result shows a great prospect of industrialization while it closely meets with the commercial demand of 10% conversion rate.

However, in the current research, most of the photoelectrode materials still need to be applied under high electrical potential (1.4–1.6 V_RHE_) to achieve a good photon–current conversion rate in the water splitting reaction [106]. The main reason can be concluded as the energy gap between the excited hole participating in the reaction from the photoanode and the HOMO state of water is too large, which hinders the excited holes from being active for OER reaction in both kinetics and thermodynamics. The general solution is to deposit a water oxidation catalyst (WOC) thin film on the photoelectrode to overcome the thermodynamic and kinetic obstacles in PEC. The common WOCs materials include the noble metal elements such as Ir, Ru, etc., but, because their expensive price and limited storage, they are not suitable for large scale industrialization; and the first-row transition metal oxides, such as Fe and Ni, can perform well in water splitting reaction with the help of high pH environment or auxiliary electrolyte [108,109,110,111,112]. The Prussian blue analog (PBA) materials, because of their special properties including simple chemical preparation and thin film deposition pathways, strong ability in remaining stable and active in a wide range of pH values, and non-toxicity, have recently received widespread attention as an anode material for OER reaction [109,111,112]. Although there are not many experiments conducted so far in exploring the role of PBA decorated photoelectrode, it has been found an indispensable function to accelerate the kinetics in the PEC water splitting of BiVO_4_. BiVO_4_ material has a compatible band gap (2.4–2.5 eV) and excellent stability for the water splitting reaction, but it has intrinsic shortcomings of poor charge transfer performance, slow oxygen release, and too-fast exciton recombination time which limit its performance in the reaction. In the solar water splitting reaction, the electron–hole recombination time τ_rec_ must be greater than the required reaction time τ_ox_ for the holes to react with water to ensure the highly-effective operation of the reaction. Conventionally, the nanostructured BiVO_4_ produced by the electrodeposition method has a τ_rec_ range in the orders of from µs (microseconds) to s (seconds), and a τ_ox_ range in the order of s range, which is undoubtedly unfavorable for the water splitting reaction [113]. This phenomenon can be attributed to the hole traps formed by the oxygen defect site O_def_ in BiVO_4_ bulk [114]. Although the energy of some traps is low (shallow traps) and is closer to the conduction band, allowing the hot charges to hop out of these traps, which enhances the solid charge conductivity to a certain extent, but for the traps with high-energy levels (deep traps), they can directly prevent the holes from hopping out, thus losing the chance to participate in the surface reaction [115], resulting in a destructive performance in the reaction of dramatically increased electron–hole recombination rate. According to this trade-off, the use of suitable WOCs to passivate the surface of BiVO_4_ can minimize the recombination of electron–hole pairs while ensuring that its conductivity is not excessively affected.

**Figure 12:**
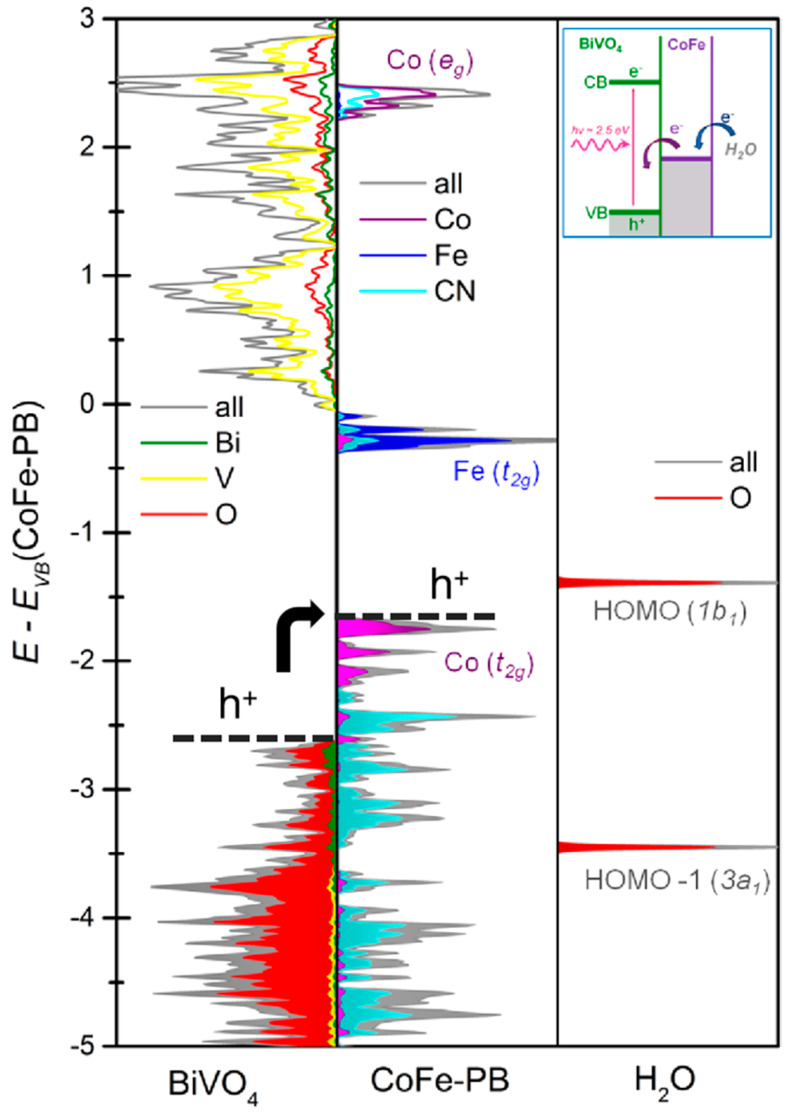
Densities of States (DOS) of BiVO_4_ (left), CoFe-PB (middle), and solvated H_2_O molecule (right) aligned by their O 2s bands. The zero-energy level is set based on the highest valence state of CoFe-PB, and the occupied electronic states are represented by filled areas. The dash lines in the picture represent the highest energy values near each material’s valence states. The curved arrows between the valence band of BiVO_4_ and CoFe-PB illustrate the preferable transfer pathways of holes induced by the co-catalyst effect of CoFe-PB. A simplified representation of electrons transfer trend is given as the inset (top right). After the qualified light illumination (>2.5 eV), BiVO_4_ generates holes (h^+^) and accumulates them in the bulk. Then, some of the holes transfer to CoFe-PB as the energy gradient. The electrons from H_2_O can diffuse to both BiVO_4_ and CoFe-PB under the same energy gradient to initial the water splitting reaction. Adapted with permission from [116]. Copyright © 2017 American Chemical Society.

Based on this principle, Hegner et al. firstly electrodeposited a CoFe-PB thin film as the competitive catalyst on the surface of BiVO_4_ photoanode in 2017 [116]. They found that the decoration of CoFe-PB can greatly decrease onset potential of the reaction by ~0.8 V (0.3 V vs. RHE) and increased the photovoltage by 0.45 V. In addition, the intensity of photocurrent (at 1.23 V vs. RHE) was remarkably enhanced by six times, and an outstanding working stability under light up to 50 h was observed. What is more, they found the different functions of CoFe-PB with the other WOCs used in the past in the water splitting reaction. In previous studies, such as on the photoelectrodes of IrO_x_ and CoPi decorated α-Fe_2_O_3_ [117,118,119], WOCs mainly worked as a capacitive layer to accumulate charges, while CoFe-PB not only directly participated in the PEC water splitting as a real co-catalyst, but also passivated the surface of BiVO_4_ bulk to minimize the probability of electron–hole recombination at the interface, and the charge transfer efficiency η_CT_ of BiVO_4_ in the experiment was exceptionally increased from 15% to 80%. They further used DFT to reveal a possible explanation of enhancement induced by CoFe-PB in water splitting reaction, the ingenious gradient energy lift formed by CoFe-PB, BiVO_4_, and water, as shown in Figure 12 [116]. It can be clearly observed in Figure 12 that the top of the valence band in BiVO_4_ and the Co (t_2g_) orbits in CoFe-PB, as well as the HOMO of water formed a gradient energy lift, which successfully reduces kinetic hindrance during the progress of holes participated in the water splitting.

**Figure 13:**
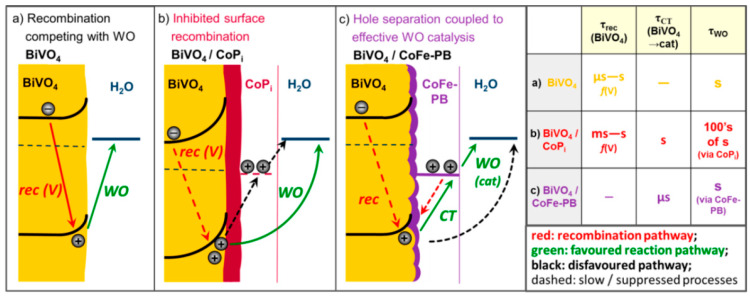
(**a**) Photon conversion efficiency in unmodified BiVO_4_ is limited by the kinetic competition between potential dependent surface recombination (rec (V)) and WO; (**b**) CoPi modification reducing the applied potential needed to effectively suppress surface recombination, allowing holes to react via the BiVO_4_ surface (Hole charge transfer (CT) to CoPi and WO via CoPi is slow and does not contribute to the photocurrent.); and (**c**) CoFe-PB enhancing the efficiency via a different mechanism. (Efficient hole CT to CoFe-PB separates holes from electrons in the BiVO_4_ surface thereby suppressing this recombination pathway. This is coupled to effective water splitting catalysis.) The table on the right reveals the varied time required for the above three materials to participate in each stage (τ_rec_ = time for BiVO_4_ electron–hole recombination time, τ_CT_ = time for charge transfer from BiVO_4_ to the co-catalyst, and τ_WO_ = time for total water oxidation) of the water oxidation. WO on CoPi is 2−3 orders of magnitude slower than WO via the BiVO_4_ surface or WO via CoFe-PB. This is mainly because the thick (100 nm) CoPi layer does not have the ability to catalyze directly, which prevents the holes in BiVO_4_ from directly reacting with water. Copied with permission from [113]. Copyright © 2018 American Chemical Society.

In the same year, this group used DFT and experiments to verify that there was no enhancement in water splitting of CoFe-PB for Fe_2_O_3_ due to the energy levels mismatch of CoFe-PB and Fe_2_O_3_. This result clarified that the rational design of a gradient energy lift between PEC semiconductor and PBA is the key to using CoFe-PB as a co-catalyst to strengthen the performance [120]. Thereafter, Shaddad et al. used NiFe-PB as a precursor to uniformly coat CoFe-PB thin film on a Zr^4+^-doped BiVO_4_ photoanode. They found that this method effectively increased the photocurrent tenfold (1.23 V vs. RHE), and obtained a low onset potential of 0.208 V (vs. RHE), while the η_CT_ significantly increased from 20% to 90% [121]. Meanwhile, Moss et al. analyzed the fast charge transfer (ms scale) mechanism of CoFe-PB for BiVO_4_ by using time-resolved absorption spectroscopy, and used CoPi and CoFe-PB decorations for comparison. In the experiment, they used photo-induced absorption spectroscopy (PIAS) to study the hole accumulation status of different materials under high voltage bias, proving that CoFe-PB and BiVO_4_ have the same function as a catalyst to consume the excited holes for water splitting reaction, while CoPi only acts as a capacitor layer to accumulate holes. Thus far, the enhancement mechanism of CoFe-PB to BiVO_4_ can be attributed to the rapid (μs or faster) hole transfer from BiVO_4_ to CoFe-PB. This charge transfer can generate long-life photo-induced holes (“Co-Fe”^2+^) without strong anode bias. Although BiVO_4_ holes are very oxidizing, the water splitting kinetics on BiVO_4_ and CoFe-PB are within a few seconds. Thus, later, the transferred photo-generated holes in CoFe-PB can directly participate in water spitting, extraordinarily reducing the waste of absorbed solar energy from electron–hole recombination. This inhibition of recombination loss is the reason for the significant lowering of the onset potential of BiVO_4_ after CoFe-PB deposition. The kinetic paths and values of bare BiVO_4_, CoFe-PB decorated BiVO_4_, and CoPi decorated BiVO_4_ are shown in Figure 13 [113].

After that, Ghobadi et al. used angular deposition method to form a heterojunction between gold and PBA-decorated BiVO_4_ [115]. This design aimed to combine the absorption effect of localized surface plasmonic resonances (LSPRs) of gold nanoparticles and the Farby–Perot (FP) modes induced by gold and BiVO_4_ to enhance the total absorption of photoelectrode in a wide spectrum (FP modes mainly enhance for the light ʎ < 520 nm and LSPRs for the light ʎ > 520 nm). At the same time, the hot holes generated from the gold nanoparticles can be used to inject into the BiVO_4_ bulk, which increases the probability of holes hopping out of the shallow trap and increases the conductivity. Under the joint action of Au and PBA, the impedance of bare BiVO_4_ was reduced from 1280.1 to 553.8 Ω, and the photocurrent (at 1.23 V vs. RHE) was increased from 190 to 1800 μA cm^−2^. This experiment shows that PBA can be used as a basic decorated method of BiVO_4_ to combine with other materials/designs to enhance the PEC water splitting performance.

Overall, it could be seen that the existence of PBA effectively lessens the recombination of photogenerated electrons and holes, thus improving the conversion efficiency of solar energy. This cognition provides a direction for further design of light absorbent materials.

## 4. Conclusions

The presence of PBA suggests novel strategies for the design of sustainable catalysts. The variable composition and structure gift PBA fascinating properties. In the application of OER in both water electrolysis and photoelectrolysis, PBA has unparalleled catalytic abilities. More specifically, for the OER in water electrolysis, PBA itself could be modified by various strategies, which could be divided into three directions: (1) doping with electron conductive nanoparticles or substrates; (2) chemically decorations including composition adjustment and surface engineering; and (3) being combined with unique structures. The common purposes of these approaches are obtaining higher charge conductivity, more exposure of active sites, and better stability. However, more effort is still expected for enhancements in these aspects.

Besides, PBA also has an irreplaceable role in acting as the precursor for other talented catalytic materials. One of the most significant intrinsic advantages of PBA is that it can be easily converted into metal oxides, phosphates, sulfides, and other compounds with OER catalytic activity. In this prospect, the main functions of PBA are as follows: (1) to help the catalytic active substances better dispersed on specific substrates; (2) to connect multiple active substances with strong chemical bonds or by the ingenious structures to achieve the synergic effect; and (3) to obtain talented configurations, such as porous structure, hollow structure, low-dimensional structure, defect-rich structure, or the combination of the above. Based on such effects, the fabricated materials are assumed to have more effective electronic structures, higher exposure rates of the active substances, and enhanced performances of electric and corrosion resistivity. Furthermore, PBA is expected to act as the bridge between more catalytic active substances and unique structures in the future.

As for the photoelectrolysis of water, the significance of PBA is mainly demonstrated through designing reasonable energy level lifts between PBA and the semiconductor, so that PBA can guide the electron transfer and thus minimize the recombination of photo-generated electrons and holes. PBA could thereby co-catalyze the water splitting reaction with semiconductors. Although bias is still required for photoelectrolysis of water, the existence of PBA provides a new idea for further design of more rational semiconductors.

In summary, although the application of PBA in catalyzing OER has been explored comprehensively, it has still a long way to go before it entirely replaces the commercial OER catalysts. Additionally, multifunctional electrodes with the capacity to catalyze OER, HER, and ORR are also expected [122]. More importantly, PBAs are constituted by Earth-abundant elements, and its application in the catalysis is conducive to the realization of sustainable catalysis for the production of clean energy. Meanwhile, the in-depth understanding of the application of PBA in OER catalysis would provide some stages for its further development or design of other green catalysts.
